# Genomic Diversity Analysis Reveals a Strong Population Structure in *Histoplasma capsulatum* LAmA (*Histoplasma suramericanum*)

**DOI:** 10.3390/jof7100865

**Published:** 2021-10-15

**Authors:** Fernando Almeida-Silva, Marcus de Melo Teixeira, Daniel R. Matute, Marcela de Faria Ferreira, Bridget M. Barker, Rodrigo Almeida-Paes, Allan J. Guimarães, Rosely M. Zancopé-Oliveira

**Affiliations:** 1 Laboratório de Micologia, Instituto Nacional de Infectologia Evandro Chagas, Fundação Oswaldo Cruz-INI/Fiocruz, Rio de Janeiro 21040-360, Brazil; marcela.ferreira@ini.fiocruz.br (M.d.F.F.); rodrigo.paes@ini.fiocruz.br (R.A.-P.); 2 Faculdade de Medicina, Universidadede Brasília, Brasília 70910-900, Brazil; marcus.teixeira@gmail.com; 3 Pathogen and Microbiome Institute, Northern Arizona University, Flagstaff, AZ 86011, USA; bridget.barker@nau.edu; 4 Biology Department, University of North Carolina, Chapel Hill, NC 27599, USA; dmatute@email.unc.edu; 5 Serviço Ambulatorial do Instituto Nacional de Infectologia Evandro Chagas, Fundação Oswaldo Cruz-INI/Fiocruz, Rio de Janeiro 21040-360, Brazil; 6 Departamento de Microbiologia e Parasitologia, Instituto Biomédico, Universidade Federal Fluminense, Niterói, Rio de Janeiro 24210-130, Brazil; allanguimaraes@id.uff.br

**Keywords:** histoplasmosis, *Histoplasma suramericanum*, genetic variation, population structure, symptoms

## Abstract

Histoplasmosis is a severe mycotic disease affecting thousands of immunocompetent and immunocompromised individuals with high incidence in Latin America, where the disease agents are *Histoplasma capsulatum* and *Histoplasma suramericanum*. In this work, we used whole-genome sequencing to infer the species diversity and the population structure of *H. suramericanum* in South America. We find evidence for strong population structure and little admixture within the species. Genome-level phylogenetic trees indicate the existence of at least three different discrete populations. We recovered the existence of a previously identified population, LAmB, and confirm that it is highly differentiated along the whole genome. We also find that *H. suramericanum* is composed of two populations, one in Northern South America, and another in the southern portion of the continent. Moreover, one of the lineages from the southern population is endemic to Rio de Janeiro and there was no association with clinical data and species isolated from patients with histoplasmosis. Our results point out the need to characterize the symptomatology of histoplasmosis caused by different species and lineages of *Histoplasma* spp.

## 1. Introduction

Histoplasmosis is one of the most prevalent endemic fungal diseases and occurs in all continents in tropical and sub-tropical areas of the globe [1]. Samuel Darling described the first case of this disease in 1906. In Latin America, the disease is responsible for thousands of deaths in immunocompromised patients, primarily due to the high burden of HIV/AIDS disease in this region; however, it is a neglected disease due to the lack of accurate diagnostic tests and notification [2]. The disease also affects immunocompetent hosts, as acute infections acquired from the environment are often associated with mining and speleology activities, fowl roosts, old building demolitions and cleaning of contaminated buildings in urban areas, or disturbing soils containing a high load of the fungus growing on bat guano or bird droppings [3]. *Histoplasma* is a dimorphic fungus and, under specific environmental conditions or in laboratory cultures at 25 to 30 °C, develops long hyphae that produce infectious microconidia and tuberculate macroconidia. The infection caused by these fungi initiates with the inhalation of airborne microconidia or hyphal fragments that, upon internationalization by alveolar macrophages, switch their morphology to single-budding yeasts, constituting the parasitic form of the fungus [3].

Natural infection often leads to an asymptomatic disease, or mild and self-resolved pneumonia in susceptible hosts, in the vast majority of the cases. Since histoplasmosis primarily affects the lungs, the acute pulmonary disease is commonly reported as Community-Acquired Pneumonia (CAP), including outbreaks involving several people due to high environmental fungal exposure [4]. If not well-managed, histoplasmosis can progress into a chronic pulmonary disorder and disseminate to different parts of the body.

Histoplasmosis is highly endemic in Latin America, especially in Brazil. The prevalence of natural *Histoplasma* spp. infections in Brazil is about 20%, but the annual incidence of histoplasmosis among people living with HIV (PLWH) is estimated to be as high as 79%, being latterly considered an AIDS-defining illness [2]. Within this population, especially for individuals with a severe immunosuppressive condition and frequently not adept to highly active antiretroviral therapy (HAART), the infection may disseminate from the lungs to spleen, liver, and bone marrow resulting in a life-threatening illness [2]. In Brazil, the number of human histoplasmosis reported cases in the literature exceeds 3500 [5], and over 2000 AIDS-associated histoplasmosis cases have been reported up to 2016 [6]. Autochthonous histoplasmosis occurs in 21 of the 26 Brazilian states, and human outbreaks have been reported primarily in the southeastern region [7]. The disease is highly endemic in São Paulo, Rio de Janeiro, Minas Gerais, and Espírito Santo, including both primary and opportunistic histoplasmosis [5]. Rio de Janeiro state (RJ) has historical importance in histoplasmosis public health studies since the first chronic and disseminated cases of this disease in Brazil were reported here [8,9]. RJ is a highly endemic area with a 93.2% prevalence of skin histoplasmin sensitivity [10]. Researchers have described at least 20 outbreaks within RJ, accounting for 50% of all Brazilian episodes reported to date [11]. Moreover, several reports of environmental, veterinarian, and human clinical isolation of *Histoplasma* have occurred over the past three decades in RJ, supporting claims of high endemicity [5]. Lastly, AIDS-associated histoplasmosis has been extensively diagnosed within southeast Brazil, suggesting RJ as a histoplasmosis hotspot in Brazil [12,13].

The genus *Histoplasma* is formed by a worldwide-distributed complex of species with distinct geographical patterns. The first phylogenetic report using the Multi Locus Sequencing Typing (MLST) methodology of four nuclear loci classified *H. capsulatum* into eight monophyletic clades [14]. By applying next-generation whole genome sequencing (WGS) to fungal species concepts, the *Histoplasma* genus was re-classified into four species as follows: *Histoplasma mississipiense* (formerly NAm1), *Histoplasma ohiense* (formerly NAm2), *Histoplasma suramericanum* (formerly LAm A), and *H. capsulatum* senso strictu (formerly Panama) [15]. South American *Histoplasma* has been reported to harbor extensive genetic variation [16], an observation that remains unexplored from a WGS point-of-view. In spite of the high prevalence of histoplasmosis in southern Brazil, the genetic composition of the *Histoplasma* isolates from the Brazilian Atlantic Forest, which is geographically isolated from other endemic areas of histoplasmosis, remains largely unexplored. MLST has revealed the existence of a prevalent genotype in southeastern Brazil, mainly in RJ. Clinical isolates from Southern Brazil (São Paulo state) appear to be phylogenetically related to the RJ genotype [16].

In this study, we bridge this gap by obtaining WGS data from clinical isolates from RJ. We used phylogenetic reconstruction and population genetics to understand the extent of the genetic diversity of Brazilian isolates of *Histoplasma* and compare the genetic profiles of those lineages to Colombian strains previously characterized as *H. suramericanum* [15]. In this work, we used MLST [14] of 50 clinical and environmental *Histoplasma* strains from a single research hospital in RJ and found the existence of at least five unique *Histoplasma* genotypes. We identified three patients carrying multiple genotypes, potentially suggesting dual infections. Next, we sequenced the genomes of 18 of those strains. The genomes of 14 isolates from RJ are phylogenetically divergent from Colombian *H. suramericanum* strains. These observations are consistent with strong structure between populations, or even species, from the southern and northern parts of South America. Moreover, we find that isolates from the LAm B clade of *Histoplasma* are phylogenetically unrelated to those species classified using genome-based taxonomy. We also show that in the sample studied, the RJ genotype was not associated with different symptoms in patients. Finally, we show that the RJ can be identified using a reduced MLST approach as opposed to whole genome sequencing. These results reveal the utter importance of genotyping natural populations of *Histoplasma* in Latin America as they harbor distinct genetic patterns.

## 2. Materials and Methods

### 2.1. Fungal Isolation and Culture Conditions

*Histoplasma* sp. environmental isolates were previously recovered from soil or clinical specimens from small wild mammals as described elsewhere [17]. We collected human-derived clinical specimens from different patients at the Evandro Chagas National Institute of Infectious Diseases (INI) Fundação Oswaldo Cruz (Fiocruz), Rio de Janeiro, Brazil. INI/Fiocruz is a reference health facility for infectious disease and especially the care of people living with HIV/AIDS in Rio de Janeiro and immunocompromised individuals from different parts of Brazil. Single monosporic colonies of 50 *H. capsulatum* isolates were inoculated and cultivated into Ham’s F12 medium (GIBCO), supplemented with 16 g/L glucose, 1 g/L glutamic acid, 8.4 mg/L cystine, and 6 g/L HEPES. The isolates were maintained at 37 °C for 14 days in a rotatory incubator at 150 rpm [17]. All clinical and environmental isolates are maintained and available at the INI/Fiocruz strains collection (Table 1).

### 2.2. Patient’s Information

The Human Research Ethics Committee of INI/Fiocruz approved the de-identified use of patient data in this study (CAAE 02109418.2.0000.5262). We recovered the medical records for 20 patients retrospectively from January 1989 to December 2016. For each patient, we obtained the following 15 symptoms: fever, weight loss, cough, dyspnea, abdominal pain, diarrhea, vomit, asthenia, headache, hepatomegaly, splenomegaly, adenomegaly, acute renal failure, hemorrhage, and skin lesion.

### 2.3. DNA Extraction

Yeast cells were recovered from seven-day cultures in Ham’s F12 Media, washed with PBS, and macerated with a pestle and mortar in liquid nitrogen. Then, the resulting fine powder was transferred (~500 mg) to a tube containing zirconia beads and 500 µL of lysis buffer (100 mM Tris pH 8; 50 mM EDTA; 1% SDS). The cell suspension was vigorously mixed (3 cycles of 30 s in a mini-bead-beater™ (Biospec Products, Bartlesville, OK, USA) and pelleted-down by centrifugation at 14,000× *g* for 2 min. To separate proteins from DNA, 500 µL of phenol:chloroform:isoamyl-alcohol (25:24:1) was added. The aqueous phase was transferred to a new tube, precipitated with isopropanol, washed with 1 mL of ethanol, and 100 µL of RNase (Sigma-Aldrich, MO, USA) was added to prevent RNA contamination. The DNA was re-suspended in 50 µL MilliQ water and integrity was determined by electrophoresis on a 1% agarose gel and quantified by spectrophotometer (GE Healthcare, Buckinghamshire, UK) as previously described [17].

### 2.4. Whole Genome Sequencing and SNP Variant Calling

Whole Genome Sequencing (WGS) was performed for 18 strains, both environmental and clinical (Table 1). To generate sequencing libraries, 1 µg of genomic DNA was processed with a KAPA Library Preparation Kit for Illumina^®^ Sequencing Platforms (Roche, Basel, Switzerland) following the manufacturer’s instructions. Equivalent amounts of paired-end libraries previously quantified using KAPA Library Quantification Kit (Kapa Biosystems) were pooled together and sequenced on a HiSeq 2500 Instrument using the v.3 chemistry on a 2 × 101 bp mode. Samples were de-multiplexed according to the corresponding barcode and initial read verification quality was accessed using FastQC [18]. The Illumina sequencing adapters were removed using the Trimmomatic v 0.36 [19]. Next, the read files to the *H. mississipiense* strain NAm 1 (AAJI00000000.1) reference genome or the *Paracoccidioides brasiliensis* strain Pb18 (ABKI00000000.2) were aligned using BWA—v 0.7.7 [20]. Potentially spurious intervals were identified using the RealignerTargetCreator and IndelRealigner modules available in the GATK v 3.3-0 [21]. Next, SNPs were called using the GATK UnifiedGenotyper [21] following the same parameters and filters previously used for polymorphism identification in *Histoplasma* species [15] as follows: QD = 2.0 || FS_filter = 60.0 || MQ_filter = 30.0 || MQ_Rank_Sum_filter = −12.5 || Read_Pos_Rank_Sum_filter = −8. Finally, Nucmer was used to identify and remove SNPs in duplicated regions and those with less than 10× coverage or with less than 10% of variant allele calls [22,23]. All .fastq files were deposited at the Sequence Archive Repository under the following accession: PRJNA497408.

### 2.5. Phylogenetic Trees Using Whole Genomes

Phylogenetic trees were generated to assess the genealogical relationships between the *Histoplasma* isolates. Then, a tree was built using the SNP dataset which included whole-genome data for 49 isolates. The Maximum Likelihood (ML) was used as implemented in IQ-TREE [24] and the -m MFP option (ModelFinder) was used for model selection [25]. To measure branch support, 1000 ultrafast bootstraps were performed and a Shimodaira–Hasegawa-like approximate likelihood ratio test (SH-aLRT) [26,27]. Phylogenetic trees were visualized with FigTree v1.4 (http://tree.bio.ed.ac.uk/software/figtree/, accessed on 17 September 2021).

### 2.6. Population Structure and Admixture

The most likely clustering scenario in South American *Histoplasma* was also studied. Two different and complementary approaches were used: first, a Principal Components Analysis (PCA) was generated using the R package *adegenet* [28]. The functions fasta2genlight and glPca were used to compute the principal components (PCs); the resulting Eigenvalues were used to generate a Neighbor-Joining (NJ) tree. Additionally, ADMIXTURE [29] was used to infer the individual ancestry of each South American isolate by comparing whole genome allele frequencies from different strains from southeastern Brazil to Colombia [15]. Polymorphisms were considered unlinked under the admixture model. Then, two lines of evidence for two populations (see Results) were found. Thus, the extent of admixture in each individual using ADMIXTURE and conditioning to two populations (K = 2) was evaluated. Individual single-color plots were used to represent homozygous lineages, whereas mixed-color bar plots represent potentially admixed genotypes; the proportion of each allele was represented as percentages.

### 2.7. Clinical Differences between RJ and Non-RJ Samples

Next, whether the RJ strains were more likely than other genotypes found in Brazil to cause any of the 15 symptoms in the clinical history of the patients was evaluated (Table 2). Additionally, the frequencies of clinical symptoms of patients infected by different *Histoplasma* genotypes (RJ vs. non-RJ) using 2-sample tests for equality of proportions with continuity correction (function *prop.test*, R package: *stats* [30]) was also compared. The power for each of these comparisons was calculated using the function pwr.2p2n.test (R package: pwr, [31]. P-values were then corrected with Bonferroni corrections as implemented in the function *p.adjust* [30].

### 2.8. Strain Genotyping Using Multi Locus Sequencing Type (MLST) Analysis

Finally, whether the RJ lineage could be detected with a MLST strategy instead of a WGS approach was also evaluated. The partial DNA sequences of four nuclear genes (*arf*, *h-anti*, *ole*, and *tub*) that have been previously used to characterize the genetic diversity of the genus [14] were used. Each sample’s DNA (extracted as described in 2.3) was amplified using PCR in 50 µL reactions containing 100 ng of a genomic DNA, 0.45 mM of each primer, 1.0 U of Taq platinum DNA polymerase (Invitrogen), 1X PCR buffer, 1.5 mM MgCl_2_, 50 mM KCl, and 0.2 mM dNTPs. The PCR had 32 cycles of DNA denaturation for 15 s at 94 °C, annealing for 30 s, and extension for 1 min at 72 °C, followed by a final extension for 5 min at 72 °C. The annealing temperature was set to 65 °C in the first cycle and reduced 0.7 °C per cycle for the next 12 cycles. Next, the PCR was continued using an annealing temperature of 56 °C for the remaining 20 cycles [21]. Additional sequences for the same four loci of 238 isolates from previously published reports were also obtained [16,17]. The DNA partial sequences were individually aligned for each locus using the MAFFT online service tool, and concatenated in the following order: *arf*, *h-anti*, *ole*, and *tub*. The final DNA matrix had 1600 bp and 288 isolates, after removing duplicate sequences. We used this dataset to build a tree following a similar approach to that described in 2.5 to generate whole genome phylogenetic trees.

## 3. Results

### 3.1. Phylogenomic Diversity

**Figure 1:**
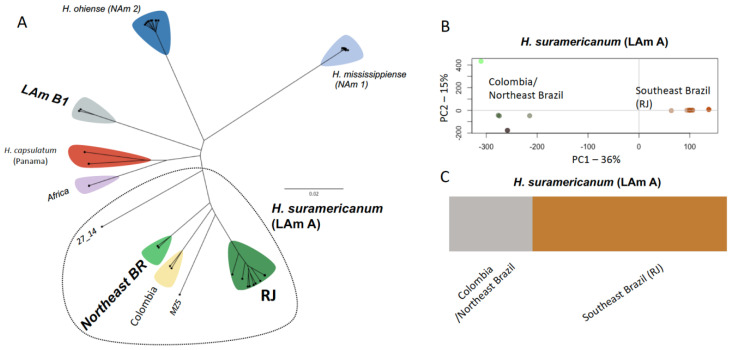
Genomic diversity of *Histoplasma* sp. collected from a single Research Institute in Rio de Janeiro/Brazil. (**A**) Unrooted Maximum Likelihood tree based on 1,470,938 SNPs indicating the existence of three genotypes: RJ, Northeast BR, and LAm B1. (**B**) Principal coordinate analysis (PCA) showing the population structure of *H. suramericanum*. The PC1 axis, which corresponds to 36% of the total genetic variation, discriminates the isolates from RJ (Southeast Brazil) and Colombia/Northeast Brazil. (**C**) Admixture analysis of *H. suramericanum* showing strong population between isolates from RJ and Colombia/Northeast. No admixture was observed between those two main groups of *H. suramericanum*.

The genomes of 18 *Histoplasma* strains were obtained and aligned to the reference *H. mississippiense* NAm 1 or the *P. brasiliensis* Pb18 genomes. Using this dataset, the consensus Maximum Likelihood tree under the GTR+F+ASC+R5 nucleotide substitution model using no outgroup or under the TVM+F+ASC+R2 model on the dataset rooted with *P. brasiliensis* was estimated (Figure S1). The unrooted phylogenomic tree suggests that *H. suramericanum* (former LAm A clade) is composed of multiple distinct phylogenetic clades: RJ, Northeast Brazil, and Colombia, suggesting local genetic variation within this species in South America. RJ, Colombia, Northeast Brazil (BR), as well the MZ5 and 27_14 lineages share a common ancestor and are limited to the South American continent (Figure 1A). This genome-wide survey also revealed that a clade previously identified with MLST and known as LAm B [14,16] is a monophyletic group. The strains IPEC 11_12 and INI 03_16 are representative of this lineage (Figure 1A and Figure S1). This clade differs from *H. suramericanum, H. capsulatum,* Africa clade, and the North American *Histoplasma* species *H. ohiense* and *H. mississippiense* (Figure 1A).

### 3.2. Population Structure within H. Suramericanum

Next, principal component analysis (PCA) was used to investigate the population structure within *H. suramericanum* (Figure 1B). PC1, which corresponds to 36% of the total variation, separated individuals from RJ (Southeast Brazil) clustered together and differed from a second cluster formed by Colombia and Northeast Brazil isolates. PC2 (15% of the total genetic variation) revealed a similar pattern, and Colombian isolates appeared differentiated from Brazilian ones. Following this two-population split, evidence for admixture between them were studied using ADMIXTURE [29]. Notably, when conditioning two lineages (K = 2), there was a recapitulation of the results from the PCA and identification of two clusters, one from Southern South America (RJ—Southeast Brazil) and one from Northern South America (Northeast Brazil and Colombia). No evidence for admixture between these groups was found (Figure 1C). These results suggest a strong population structure within *H. suramericanum* between northern and southern parts of South America and pose the possibility of *Histoplasma* speciation within the South American continent.

Given the existence of the lineages of *Histoplasma* in South America, differences in the clinical manifestations of histoplasmosis caused by different lineages were evaluated. For the statistical analysis, we excluded three patients with two distinct genotypes isolated. The medical records of the others 17 patients included in this study were accessed, and information was drawn regarding differences in the prevalence of 15 different clinical features of histoplasmosis. Ten of these patients were infected by the RJ genotype and seven by other genotypes. Table 2 shows the 15 signs and symptoms. We refrained from conducting comparisons with low power (power < 0.4, Table 2). Out of the four symptoms (diarrhea, asthenia, headache, and acute renal failure) with enough power for pairwise comparisons, all were equally prevalent in histoplasmosis caused by RJ and other lineages. The other characteristics showed no difference between the RJ clade and the non-RJ clade. However, increasing sampling, differences in the symptoms of histoplasmosis caused by different *Histoplasma* strains may be observed. A systematic assessment of this issue with a larger number of samples is sorely needed. A systematic assessment of this issue with a larger number of samples is sorely needed.

### 3.3. MLST Is Sufficient to Identify the RJ Clade

**Figure 2:**
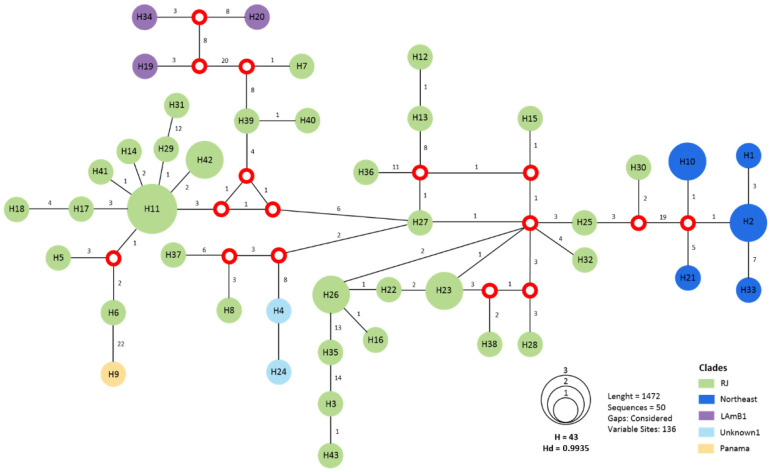
Median vector haplotype network demonstrating the phylogenetic relationships among the 50 isolates of the present study grouping both in RJ clade and others. Red dots represent median vectors. Circle size is proportional to the frequency of isolates (h). The numbers around each vertex represent the number of mutations separating each haplotype.

Finally, we studied whether the RJ lineage and other South American *Histoplasma* lineages could be identified using MLST. This approach can be advantageous because of its cost-effectiveness and rapidity. Our MSLT dataset suggests that the majority of the strains from Rio de Janeiro (*n* = 34) grouped in a cluster separated from the Northeast clade by 19 mutations, from LAm B by 22 mutations, and from *H. capsulatum sensu stricto* (i.e., Panama) by 22 mutations (Figure 2). Conversely, this MLST scheme allows to uniquely identify each of these other three lineages. Even though MLST is limited to infer the evolutionary processes involved in pathogen diversification [32], our results indicate that this approach is efficient to differentiate between South American lineages of *Histoplasma*.

### 3.4. Multiple Infections Caused by Histoplasma spp.

Our MLST screening also allowed us to determine whether each patient was infected by more than one haplotype. Five patients had at least two *Histoplasma* isolates during the course of the study. Two patients, numbers 8 and 20, carried two isolates with the same background as follows: LAm B and RJ. Three patients were infected by strains with different genetic backgrounds. Patients 1 and 9 had both RJ and Northeast genotypes, while patient number 13 was infected with the RJ and Unknown *Histoplasma* genotypes.

## 4. Discussion

Studies aiming to explore the genetic complexity of *H. capsulatum* sensu lato began around the year of 1990, and at least 17 phylogenetic lineages are recognized so far [16,33,34]. Four of those phylogenetic lineages were recently reclassified into different species based on genome congruence criteria [15]. Despite the evidence for strong genetic isolation, interspecific admixture has also been reported in *Histoplasma.* Gene flow can increase the possibility of the emergence of new strains with varied clinically relevant phenotypes [35,36]. For example, different levels of virulence and different strategies of host immune system escape have been proposed for different species of *Histoplasma* [37,38]. Thus, understanding the phylogenetic relationships, population genetics, and epidemiology of *Histoplasma* is essential for investigating local disease dynamics, outbreaks, pathogenicity, and drug resistance. It is worth noting that most of the genetic diversity of *Histoplasma* is observed in Latin America, and molecular epidemiological studies are urgently needed. Our results have three major implications for our understanding of the biology of *Histoplasma* in South America: (i) the existence of a clade, RJ, that is prevalent in southern Brazil, (ii) the strong population structure of *H. suramericanum* which is formed by at least two well-defined populations, and (iii) the high genetic diversity of *Histoplasma* in South America. We discuss each of these implications in the sections that follow.

### 4.1. Histoplasma Genotypes from Southeast Brazil, Rio de Janeiro

Both of our genotyping surveys, genome-wide and MLST, suggest the existence of a Rio de Janeiro (RJ) population, which is part of the previously proposed LAm1 MLST clade [16]. This genotype has been identified in all environmental and animal *Histoplasma*-derived strains from this state, suggesting that human cases of histoplasmosis caused by the RJ genotype in Rio de Janeiro are locally acquired. So far, 58 strains have been typed as the RJ genotype and in its majority are composed by isolates from Southeastern Brazil, which suggests a recent emergence or a low ability for migration (Figure 2, [14,16]).

Notably, histoplasmosis caused by RJ and non-RJ clades was not associated with any clinical signs and symptoms. However, it is important to note that some limitations of this work are the low number of patients infected by different genotypes (i.e., low statistical power for pairwise comparisons) and the fact that this is a single-center study. In addition, as the center where the study was conducted is focused to treat patients living with HIV/AIDS, the number of *Histoplasma* isolates from non-HIV infected patients in this study is insufficient to address possible associations between genotypes and histoplasmosis manifestations in patients with or without HIV infection. Additionally, we detected dual infections with RJ and other genotypes (Northeast, Lam B, and Unknown). This pattern has already been observed by our research group in HIV/AIDS patients from Northeast Brazil [17]. These dual infections could be due local infections by multiple genotypes or by the reactivation of a previous latent *Histoplasma* infection because of the immunosuppressive condition of patients living with HIV/AIDS. It is worth mentioning that these patients have a migratory history reported. Nonetheless, this observation deserves to be followed up with larger studies. Clinical and genomic researchers need to collaborate to study the symptomatology caused by different species and populations of *Histoplasma*. Multicenter studies involving large cohorts from different geographic regions and, ideally, genome sequencing, are necessary to fully understand the impact of *Histoplasma* genotypes in the clinical setting.

### 4.2. Strong Population Structure in H. suramericanum in South America

Previous MSLT studies suggested a complex structure within the *Histoplasma* LAm A phylogenetic species (now formally known as *H. suramericanum*). Both phylogenetic and population genetic studies herein conducted also show a strong partition of the genetic variation within this clade. Phylogenomic analysis revealed the presence of two main groups composed by strains collected in both northern and southern parts of South America, indicating a strong geographical split within *H. suramericanum*. In this effort, we focused on the *H. suramericanum* RJ population which seems to be endemic to the Brazilian Atlantic Forest. This biome is a biodiversity hotspot for plants [39], birds (e.g., spinetails) [40], and mammals [41]. Nonetheless, no effort has determined whether the Atlantic Forest also harbors more fungal pathogens. Future studies should explore the possibility that cosmopolitan fungal pathogens show local adaptation and speciation when associated to particular biomes.

Other endemic human fungal pathogens also show evidence of genetic differentiation within the American continent. *Paracoccidioides brasiliensis* is predominately found in the southern part of South America, while *P. restrepiensis* and *P. venezuelensis* are found in Northern part of the continent. A strong genetic bottleneck was observed within *Coccidioides posadasii* by comparing clinical isolates from Central/South America to Mexico and the USA. Similarly, *Sporothrix schenckii* isolates from North America and South America are genetically different from each other. All these reports have been somehow limited by the availability of clinical and environmental samples. Larger genomic surveillances of endemic fungal pathogens and synergy between clinicians and fungal geneticists are needed to deeply understand the evolutionary history of these pathogens.

### 4.3. South America Harbors at Least Two Different Species of Histoplasma

Genome sequencing has revolutionized the definition of species boundaries in fungi (reviewed in [42]). Pathogenic fungi are on the rise since taxonomy changes over time by incorporating more taxa from unexplored areas and due to the implementation of different methods. Moreover, researchers and clinicians claim for the correlation of divergent species with a plausible clinically relevant phenotype (i.e., disease manifestation, antifungal resistance, antigenic variation). The pioneer studies aiming to quantify the genetic magnitude of *Histoplasma* in the American continent have proposed multiple genotypes that were latter classified into former species as follows: *H. capsulatum* senso strictu (Panama), *H. mississipiense* (NAm1), *H. ohiense* (NAm2), and *H.* *suramericanum* (LAm A) [15]. A fifth phylogenetic species, named LAm B was also described using MLST data, showing strong genetic isolation from other Latin American and North American genotypes [14]. However, the genomes of this putative phylogenetic species have never been studied. Here, we bridge that gap and show that LAm B is genetically differentiated from other *Histoplasma* species across its genome (Figure 1A and Figure S1). We do refrain from defining it as a species because the group only has two known strains, both included in this study. Previous studies suggest that this genotype is broadly distributed in South America, and its occurrence was documented in Colombia and Argentina, beyond Brazil [16,43].

More generally, our MLST genetic survey revealed the possibility of other clades, some of which might be highly differentiated species. For example, unknown1, a lineage composed by the two isolates, H4 and H24, seems to be mostly but not completely isolated from *H. suramericanum* (see H8 and H37 in Figure 2). As of now, we have no data that allows us to conclude these are different species, but the level of differentiation at the four diagnostic loci among lineages is certainly high.

## 5. Conclusions

We found evidence for strong population structure and little admixture within *H. suramericanum*. One of the lineages within the *H. suramericanum* Southern population is endemic to Rio de Janeiro and seems to have clinical impact in histoplasmosis patients. Additional genome sequencing efforts aiming to address critical gaps in the taxonomy, ecology, and evolution of this important fungal pathogen are needed in South America.

## Figures and Tables

**Table 1:** General information of the 50 studied isolates in this study.

Patient	Isolate	Origin	Specimen	Clinical Features	Phylogenetic Species
1	13H	Human	Blood Culture	HIV+/Disseminated	NE
2	129H	Human	Blood Culture	HIV+/Disseminated	NE
3	20231	Human	Tongue Scraping	HIV+/Disseminated	Unknown1
-	11354	Human	Blood Culture	HIV+/Disseminated	Unknown1
4	3416	Human	Sputum	HIV−/Chronic Pulmonary	RJ
-	3612	Human	Lung Biopsy	HIV+/Disseminated	RJ
-	39439	Human	Bone Marrow Aspirate	HIV+/Disseminated	RJ
-	39942	Human	Biopsy	NA	Panama
5	6503	Human	Ganglion Biopsy	HIV+/Opportunistic	RJ
-	84502	Human	Blood Culture	HIV+/Disseminated	NE
-	84564	Human	Skin Biopsy	HIV+/Disseminated	NE
-	AC 05	Environmental	Soil	NA	RJ
-	CÃO 4	Animal/Dog	Liver and Spleen	NA	RJ
-	CO4	Environmental	Soil	NA	RJ
-	EP 02	Environmental	Soil	NA	RJ
1	HC 18	Human	Blood Culture	HIV+/Disseminated	RJ
6	40039	Human	Bone Marrow Aspirate	HIV+/Disseminated	RJ
-	IGS 19	Environmental	Soil	NA	RJ
-	IGS 4/5	Environmental	Soil	NA	RJ
7	INI 01/16	Human	Bone Marrow Aspirate	HIV+/Disseminated	NE
8	INI 02/16	Human	Skin Biopsy	HIV+/Disseminated	LAmB1
8	INI 03/16	Human	Bone Marrow Aspirate	HIV+/Disseminated	LAmB1
9	INI 04/16	Human	Sputum	HIV-/Disseminated	NE
9	INI 05/16	Human	Ganglion Fragment	HIV-/Disseminated	RJ
10	INI 06/16	Human	Bone Marrow Aspirate	HIV+/Disseminated	RJ
11	INI 07/16	Human	Bone Marrow Aspirate	HIV+/Disseminated	Unknown1
12	01_12	Human	Bone Marrow Aspirate	HIV+/Disseminated	RJ
13	01_13	Human	Blood Culture	HIV+/Disseminated	RJ
-	02_13	Animal/Dog	Blood Culture	NA	RJ
14	02_14	Human	Skin Biopsy	HIV+/Disseminated	RJ
15	04_12	Human	Bone Marrow Aspirate	HIV+/Disseminated	RJ
16	04_14	Human	Bone Marrow Aspirate	HIV+/Disseminated	RJ
-	05_12	Animal/Cat	Lesion Exudate	NA	RJ
17	06_12	Human	Bone Marrow Aspirate	HIV+/Disseminated	RJ
-	07_12	Animal/Dog	Lymph Node Biopsy	NA	RJ
18	09_12	Human	Bronchoalveolar Lavage	Chronic Pulmonary	NE
19	11_12	Human	Skin Biopsy	HIV+/Disseminated	LAmB1
13	23_11	Human	Blood Culture	HIV+/Disseminated	Unknown1
-	24_11	Human	Blood Culture	HIV+/Disseminated	RJ
-	26_11	Human	Bone Marrow Aspirate	HIV+/Disseminated	RJ
20	27_11	Human	Oropharyngeal Swab	HIV+/Disseminated	RJ
20	28_11	Human	Bone Marrow Aspirate	HIV+/Disseminated	RJ
-	IT 04	Environmental	Soil	NA	RJ
-	RPS 35	Environmental	Soil	NA	RJ
-	RPS 45	Environmental	Soil	NA	RJ
-	RPS 51	Environmental	Soil	NA	RJ
-	RS 36	Wild Animal	NA	NA	RJ
-	TI 01	Environmental	Soil	NA	RJ
-	TI 05	Environmental	Soil	NA	RJ
-	36GAL	Human	Blood	HIV+/Disseminated	Unknown1

Legend: NA = Not Available.

**Table 2:** Clinical features of histoplasmosis cases distributed among different genotypes of *H. capsulatum* isolated in National Institute of Infectious Diseases Evandro Chagas/Fiocruz, Brazil.

Clinical Data	Genotype		Power	*p*-Value	Corrected *p*-Value
	RJ	Others			
Fever	9 (90%)	5 (71.4%)	0.166		
Weight Loss	6 (60%)	5 (71.4%)	0.078		
Cough	6 (60%)	6 (85.7%)	0.226		
Dyspnea	3 (30%)	1 (14.2%)	0.122		
Abdominal Pain	3 (30%)	2 (28.5%)	0.050		
Diarrhea	1 (10%)	4 (57.1%)	0.584	0.119	0.476
Vomit	3 (30%)	3 (42.8%)	0.085		
Asthenia	3 (30%)	5 (71.4%)	0.411	0.234	0.701
Headache	0 (0%)	2 (28.5%)	0.629	0.301	0.701
Hepatomegaly	4 (40%)	2 (28.5%)	0.078		
Splenomegaly	5 (50%)	4 (57.1%)	0.060		
Acute Renal Failure	7 (70%)	2 (28.5%)	0.411	0.234	0.701
Hemorrhage	3 (30%)	1 (14.2%)	0.122		
Skin Lesion	4 (40%)	3 (42.8%)	0.0516		
Adenomegaly	6 (60%)	4 (57.1%)	0.0516		

## Data Availability

Not applicable.

## Supplementary Materials

The following are available online at https://www.mdpi.com/article/10.3390/jof7100865/s1, Figure S1: Whole-genome Maximum Likelihood tree of *Histoplasma* sp.
