# Supplementary material for: Genomic Diversity Analysis Reveals a Strong Population Structure in Histoplasma capsulatum LAmA (Histoplasma suramericanum)
Source: J Fungi (Basel). 2021 Oct 15;7(10):865. doi: 10.3390/jof7100865 (PMC8540737; doi:10.3390/jof7100865)
Supplement: Supplementary file 1 [file jof-07-00865-s001.zip › jof-1369034-supplementary.pdf]

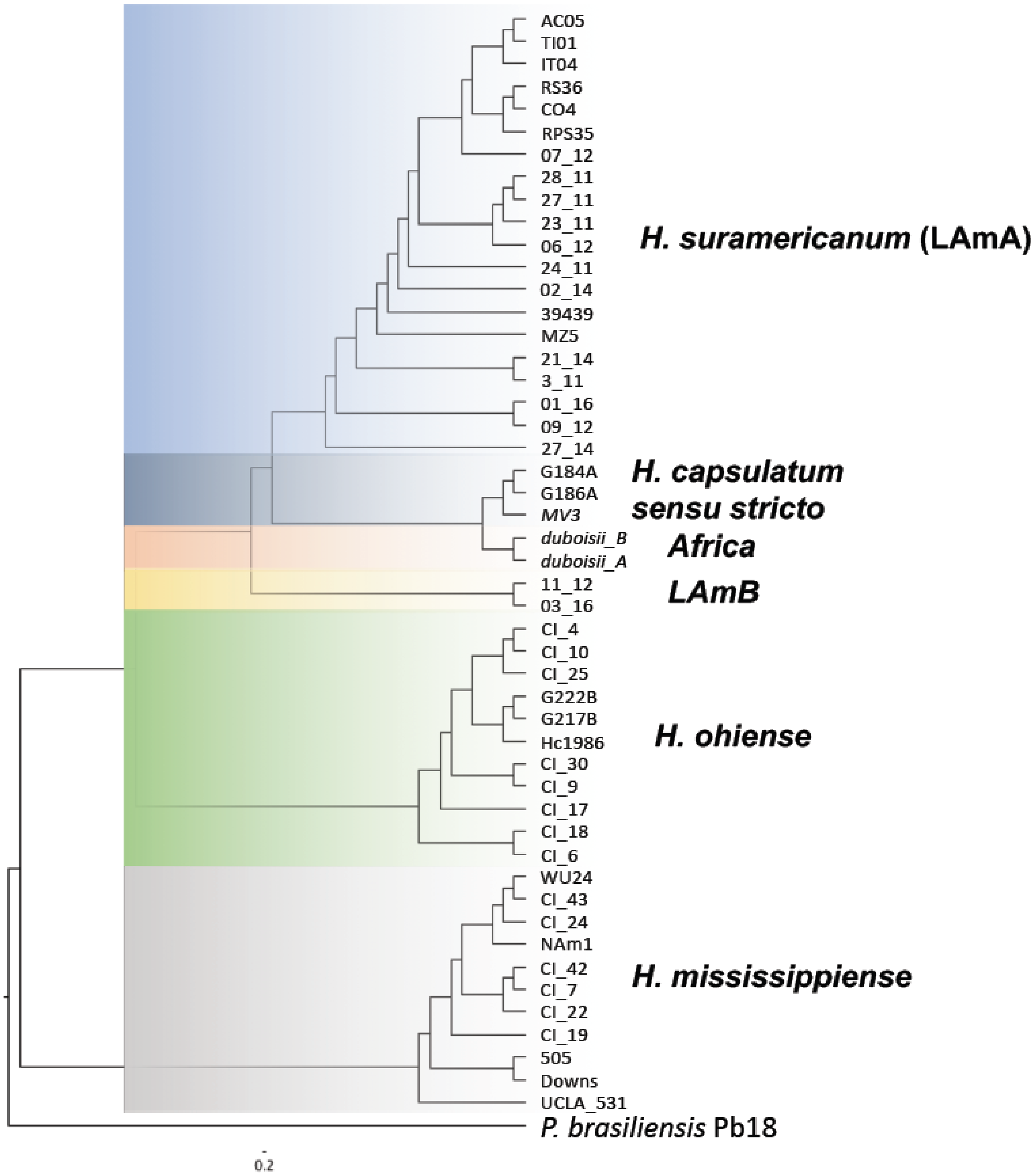

Figure S1. Whole-genome Maximum Likelihood tree of *Histoplasma* sp. The phylogenomic tree was rooted with *Paracoccidioides brasiliensis* Pb18 strain. The branches was transformed to proportional and the main clusters of *Histoplasma* are highlighted.
